# Accounting for thermodynamic non-ideality in the Guinier region of small-angle scattering data of proteins

**DOI:** 10.1007/s12551-016-0235-5

**Published:** 2016-11-22

**Authors:** David J. Scott

**Affiliations:** 1 School of Biosciences, University of Nottingham, Sutton Bonington Campus, Sutton Bonington, Leicestershire LE12 5RD UK; 2 Research Complex at Harwell, Rutherford Appleton Laboratory, Harwell Oxford, Didcot, Oxfordshire OX11 0FA UK; 3 ISIS Spallation Neutron and Muon source, Rutherford Appleton Laboratory, Harwell Oxford, Didcot, Oxfordshire OX11 0FA UK

**Keywords:** Small-angle scattering, Thermodynamic non-ideality, Guinier region

## Abstract

Hydrodynamic studies of the solution properties of proteins and other biological macromolecules are often hard to interpret when the sample is present at a reasonably concentrated solution. The reason for this is that solutions exhibit deviations from ideal behaviour which is manifested as thermodynamic non-ideality. The range of concentrations at which this behaviour typically is exhibited is as low as 1–2 mg/ml, well within the range of concentrations used for their analysis by techniques such as small-angle scattering. Here we discuss thermodynamic non-ideality used previously used in the context of light scattering and sedimentation equilibrium analytical ultracentrifugation and apply it to the Guinier region of small-angle scattering data. The results show that there is a complementarity between the radially averaged structure factor derived from small-angle X-ray scattering/small-angle neutron scattering studies and the second virial coefficient derived from sedimentation equilibrium analytical ultracentrifugation experiments.

## Introduction

The technique of small-angle scattering by X-rays and neutrons is enjoying a resurgence due to better sources and much improved data analysis. One issue with such studies concerns those carried out at semi-dilute concentrations (>2 mg/ml) where signal-to-noise ratios need to be improved and better quality data obtained. The main feature of this concentration regime is the effect of inter-particle interference whereby the molecules under study can no longer be treated as isolated particles, but exhibit some scattering between molecules. This effect can also be interpreted as thermodynamic non-ideality and as such be treated in a similar manner to that observed in light scattering and sedimentation equilibrium analytical ultracentrifugation. Exactly how this interpretation can be made has been the subject of several studies, and it is the purpose of this article to show that the two approaches are compatible.

### The effect of thermodynamic non-ideality upon angular dependence: Guinier analysis

The scattering curve is given by the relationship:

1 $$ I(Q)=cKP(Q)S(Q) $$

This can be expanded to:

2 $$ I(Q)\propto \left(4\pi {\displaystyle \underset{0}{\overset{D_{\max }}{\int }}P(r)\frac{ \sin (Qr)}{Qr}dr}\right)\left(1+4\pi {n}_p{\displaystyle \underset{0}{\overset{\infty }{\int }}\left[g(r)-1\right]\frac{ \sin (Qr)}{Qr}{r}^2dr}\right) $$

where the first term in brackets is the form factor *P*(*Q*) and the second term is the structure factor *S*(*Q*). There are constants of proportionality also implicit in Eq. 2 which pertain both to the concentration (*c*) of the sample and the contrast of the sample (*K*) with respect to whether the sample is being observed using X-rays or neutrons. As a general case is being derived here, only the proportional relationship (Eq. 2) is discussed. The first term can be expressed as an expansion in the McLaurin series and, therefore, so can the second term.

The Guinier expansion of the form factor is:

3 $$ \sin (Qr)=Qr-\frac{1}{3!}{(Qr)}^3-\frac{1}{5!}{(Qr)}^5+\dots $$

which is substituted into *P*(*Q*):

4 $$ I(Q)=4\pi {\displaystyle \underset{0}{\overset{D_{\max }}{\int }}P(r)dr}-4\pi \frac{1}{3!}{\displaystyle \underset{0}{\overset{D_{\max }}{\int }}P(r)\cdot {(Qr)}^2dr}-\dots $$

truncated at the *Q* ^2^ term. The corresponding expansion for *S*(*Q*) is:

5 $$ S(Q)=1+4\pi {n}_p\cdot {\displaystyle \underset{0}{\overset{\infty }{\int }}\left[g(r)-1\right]{r}^2dr}-4\pi {n}_p\cdot {\displaystyle \underset{0}{\overset{\infty }{\int }}\left[g(r)-1\right]{(Qr)}^2{r}^2dr-\dots } $$

Multiplying the terms in *P*(*Q*) with *S*(*Q*) and again truncating at the *Q* ^2^ term, *I*(*Q*) becomes:

6 $$ \begin{array}{l}4\pi {\displaystyle \underset{0}{\overset{D_{\max }}{\int }}P(r)dr}+4\pi {\displaystyle \underset{0}{\overset{D_{\max }}{\int }}P(r)dr}\cdot {\displaystyle \underset{0}{\overset{\infty }{\int }}\left[g(r)-1\right]{r}^2dr}\\ {}-4\pi \frac{1}{3!}{\displaystyle \underset{0}{\overset{D_{\max }}{\int }}P(r)\cdot {(Qr)}^2dr}+4\pi \cdot \frac{1}{{\left(3!\right)}^2}\cdot {\displaystyle \underset{0}{\overset{\infty }{\int }}\left[g(r)-1\right]{r}^2dr}{\displaystyle \underset{0}{\overset{D_{\max }}{\int }}P(r)\cdot {(Qr)}^2dr}-\dots \end{array} $$

Comparison of terms reveals that *I*(0) now becomes:

7a $$ I(0)=4\pi {\displaystyle \underset{0}{\overset{D_{\max }}{\int }}P(r)dr}+4\pi {\displaystyle \underset{0}{\overset{D_{\max }}{\int }}P(r)dr}\cdot {\displaystyle \underset{0}{\overset{\infty }{\int }}\left[g(r)-1\right]{r}^2dr} $$

and the *R*_g_ term becomes:

7b $$ -4\pi \frac{1}{3!}{\displaystyle \underset{0}{\overset{D_{\max }}{\int }}P(r)\cdot {(Qr)}^2dr}+4\pi \cdot \frac{1}{{\left(3!\right)}^2}\cdot {\displaystyle \underset{0}{\overset{\infty }{\int }}\left[g(r)-1\right]{r}^2dr}{\displaystyle \underset{0}{\overset{D_{\max }}{\int }}P(r)\cdot {(Qr)}^2dr} $$

Hence, as *S*(*Q*) approaches 1, the term *g*(*r*)-1 integral approaches zero. Therefore, the structure factor terms become negligible and the relationship reverts to the dilute case. Whether *R*_g_ increases or decreases consequently depends on the sign of the term *g*(*r*), i.e. the pair correlation function, at low angles. This predicts that for replusive interactions both *R*_g_ and I(0) will fall with decreasing protein concentration and that for attractive interactions, I(0) will rise with increasing protein concentration, whereas *R*_g_ will fall in the dilute regime for which the structure factor is term is smaller than the form factor term: a situation seen experimentally when measurements are made over a range of concentrations (Rubinson et al. 2008). This methodology provides a link between the intermolecular potentials and the change in *R*_g_ derived from the Guinier region. Expansion of the data further into the higher angle region is not possible in this analysis due to the linearity approximations made; however it is hoped that the methodology will prove useful in providing further stimulus to the analysis of non-ideality in small-angle scattering. In the following section I look at the thermodynamic origins of non-ideality in small-angle scattering experiments and the information that can be usefully extracted from experiments.

### Relationship with virial coefficients

The structure factor and is related to the second virial coefficient by:

8 $$ { \lim}_{Q\to 0}S(Q)=\frac{RT}{M}\cdot {\left(\frac{\partial \Pi}{\partial c}\right)}_{P,T}^{-1} $$

where *R* is the universal gas constant and *T* is the thermodynamic temperature, and where the constraints of temperature and pressure mean that we are able to expand into a set of virial coefficients on a molal basis (Zimm 1948; Stockmayer 1950).

By making the expansion:

9 $$ \Pi =cRT\left(\frac{1}{M}+{A}_2c+{A}_3{c}^2+\dots \right) $$

and then differentiating with respect to the molal concentration

10 $$ \left(\frac{\partial \Pi}{\partial c}\right)=RT\left(\frac{1}{M}+{A}_2c+{A}_3{c}^2+\dots \right) $$

we can express the structure factor as a set of virial coefficients:

11 $$ \underset{c\to 0}{ \lim }S(q)=\frac{RT}{M}\cdot {\left(RT\left(\frac{1}{M}+{A}_2c+{A}_3{c}^2+\dots \right)\right)}_{P,T}^{-1} $$

at the dilute limit this then becomes

12 $$ \underset{c\to 0}{ \lim }S(q)=1 $$

Deriving an angular dependence is straightforward using the approximation of Zimm (1948) in a more user friendly form in Receveur et al. (1998):

13 $$ \frac{I\left(0,0\right)}{I\left(Q,c\right)}=\frac{1}{P(Q)}+2{A}_2M{c}_2+3{A}_3M{c}_2^2+\dots $$

where* P*(*Q*) is the form factor, *A*_i_ is the weight/weight virial coefficient and *M* is the molecular weight of the macromolecule in solution. Then at zero angle,* P*(*Q*) = 1 by definition, hence we have:

14 $$ \frac{I\left(0,0\right)}{I\left(0,c\right)}=2{A}_2M{c}_2+3{A}_3M{c}_2^2+\dots $$

Now, the definition of osmotic pressure in terms of concentration c is:

15 $$ \varPi =RT\left[\frac{c_2}{M}+{A}_2M{c}_2^2+{A}_3M{c}_2^3+\dots \right] $$

Equation 13 is obviously the derivative of equation 15 with respect to c_2_ multiplied by a factor of M/RT

This gives the form:

16 $$ I\left(0,0\right)=\frac{RT}{M}{\left(\frac{\partial \varPi }{\partial {c}_2}\right)}^{-1} $$

This directly relates the forward scattering and to the osmotic virial coefficient analysis by Eisenberg (1976) and Winzor et al. (2006) and provides a direct link between the thermodynamic interpretation of non-ideality and interparticle interference. A molecular interpretation of this is that the virial coefficient is a thermodynamic measure of how the motion of the particles in solution are correlated with each other, and the stronger the interaction defined by the correlation function, then the further the thermodynamic behaviour of the system deviates from ideality.

### Contribution of co-solute to thermodynamic non-ideality

If the co-solute term is not considered, then according to Hill (1968) we can write the second virial term in terms of molar concentration as:

17 $$ 2M{A}_2c=2{B}_{22}c $$

where *B*_22_ is the self–self molar second virial coefficient that can be used for statistical mechanics purposes, as long as there is a conversion of the molal virial coefficients to molar. However, Winzor et al. (2007) found from light scattering that there is a non-equivalence of the second virial coefficient determined due to an additional contribution from the co-solute

18 $$ {A}_2={B}_{22}+\varOmega {c}_3 $$

Omega has previously been shown to be a function dependent upon *B*_23_, the solute and the co-solute second virial coefficient. Hence, at low concentrations, but high co-solute concentrations, *A*_2_ will still have a magnitude that is approximately proportional to *B*_23_ *c*_3_ and, therefore, *S*(*Q*) will still tend to 1 with concentration, but with a much reduced dependency on *c*. Thus, at high co-solute concentrations, such as studies of unfolded protein at 4–6 M urea, this term *B*_23_ *c*_3_ will have a considerable effect upon the nature of the scattering curve through the structure factor term (Scott et al. 2013). Such changes have previously been ascribed to aggregation, and as such great care is needed in the interpretation of non-ideal data in the Guinier region.

## Conclusions

In this paper I derive a relationship for how the Guinier region of a small-angle scattering curve is affected by structure factor information and then relate this directly to the thermodynamic non-ideality of the system through virial coefficients. In interpreting this region, great care is needed, as an inadequate account of co-solute interactions will lead to erroneous interpretation of the data. With these caveats in hand, I hope that these relationships will be of use in interpreting the data from small-angle scattering studies.
