# Erratum to: Accounting for thermodynamic non-ideality in the Guinier region of small-angle scattering data of proteins

**DOI:** 10.1007/s12551-017-0328-9

**Published:** 2017-10-23

**Authors:** David J. Scott

**Affiliations:** 1 School of Biosciences, University of Nottingham, Sutton Bonington Campus, Leicestershire, Sutton Bonington LE12 5RD UK; 2 Research Complex at Harwell, Rutherford Appleton Laboratory, Harwell Oxford, Oxfordshire, Didcot OX11 0FA UK; 3 ISIS Spallation Neutron and Muon Source, Rutherford Appleton Laboratory, Harwell Oxford, Oxfordshire, Didcot OX11 0FA UK

**Erratum to: Biophys Rev (2016) 8:441–444**

10.1007/s12551-016-0235-5

The article “Accounting for thermodynamic non-ideality in the Guinier region of small-angle scattering data of protein”, written by David J. Scott, was originally published without open access. After publication in volume 8, issue 4, page 441–444 it was found that article should be an Open Choice and as an open access publication. Therefore, the copyright of the article has been changed to © The Author(s) 2017 and the article is forthwith distributed under the terms of the Creative Commons Attribution 4.0 International License (http://creativecommons.org/licenses/by/4.0/), which permits use, duplication, adaptation, distribution and reproduction in any medium or format, as long as you give appropriate credit to the original author(s) and the source, provide a link to the Creative Commons license, and indicate if changes were made.

The original article has been corrected.

